# Incomplete immune reconstitution and its predictors in people living with HIV in Wuhan, China

**DOI:** 10.1186/s12889-023-16738-w

**Published:** 2023-09-16

**Authors:** Wenyuan Zhang, Jisong Yan, Hong Luo, Xianguang Wang, Lianguo Ruan

**Affiliations:** 1 Department of Infectious Diseases, Wuhan Jinyintan Hospital, Tongji Medical College of Huazhong University of Science and Technology, Wuhan, 430023 Hubei China; 2 Hubei Clinical Research Center for Infectious Diseases, Wuhan, 430023 Hubei China; 3 Wuhan Research Center for Communicable Disease Diagnosis and Treatment, Chinese Academy of Medical Sciences, Wuhan, 430023 Hubei China; 4 Joint Laboratory of Infectious Diseases and Health, Wuhan Institute of Virology and Wuhan Jinyintan Hospital, Chinese Academy of Sciences, Wuhan, 430023 Hubei China; 5 Department of Respiratory and Critical Care Medicine, Wuhan Jinyintan Hospital, Tongji Medical College of Huazhong University of Science and Technology, Wuhan, 430023 Hubei China

**Keywords:** HIV/AIDS, Immune reconstitution, Nomogram, Predictive model

## Abstract

**Objective:**

This study aimed to build and validate a nomogram model to predict the risk of incomplete immune reconstitution in people living with HIV (PLWH).

**Methods:**

Totally 3783 individuals with a confirmed diagnosis of HIV/AIDS were included. A predictive model was developed based on a retrospective set (*N* = 2678) and was validated using the remaining cases (*N* = 1105). Univariate and multivariate logistic regression analyses were performed to determine valuable predictors among the collected clinical and laboratory variables. The predictive model is presented in the form of a nomogram, which is internally and externally validated with two independent datasets. The discrimination of nomograms was assessed by calculating the area under the curve (AUC). Besides, calibration curve and decision curve (DCA) analyses were performed in the training and validation sets.

**Results:**

The final model comprised 5 predictors, including baseline CD4, age at ART initiation, BMI, HZ and TBIL. The AUC of the nomogram model was 0.902, 0.926, 0.851 in the training cohort, internal validation and external cohorts. The calibration accuracy and diagnostic performance were satisfactory in both the training and validation sets.

**Conclusions:**

This predictive model based on a retrospective study was externally validated using 5 readily available clinical indicators. It showed high performance in predicting the risk of incomplete immune reconstitution in people living with HIV.

**Supplementary Information:**

The online version contains supplementary material available at 10.1186/s12889-023-16738-w.

## Introduction

The use of antiretroviral therapy (ART) suppresses viral replication and increases CD4^+^ T cell counts [1–3], improving the prognosis of the majority of people living with HIV (PLWH) and dramatically decreasing both morbidity and mortality in acquired immunodeficiency syndrome (AIDS) [4, 5]. However, up to 10–40% of patients may fail to achieve a sufficient immunologic response, as assessed by CD4^+^ T cell count, despite HIV virologic suppression, and are referred to as “immunologic non responders” (INRs) [6, 7]. Compared with PLWH achieving good immune reconstitution, these patients show a greater risk of AIDS-defining diseases and non-AIDS-defining events (nADE), which is associated with high mortality [8–10].

Incomplete immune reconstitution is pathophysiologically thought to be associated with decreased bone marrow hematopoiesis, thymic dysfunction, residual viral replication, altered gut microbiota, and coinfections, particularly persistent inflammation and abnormal immune activation, significantly decreasing CD4 production and persistent CD4 destruction [7, 11–13]. Several therapeutics, e.g., immunosuppressive agents [14] and cytokines [15, 16], have been used to limit and restore chronic insufficient immune reconstitution for a long time, however, with marginal success.

To date, no effective treatment could recover CD4^+^ T cells, especially in INRs. At present, it is particularly important to assess patient condition earlier, especially at the initial examination, and to adopt a timely and individualized treatment plan. It is commonly admitted that several factors can predict immunological function recovery and disease progression, e.g., CD4^+^ T cell count, CD4/CD8 ratio, viral load (VL) and IFN-γ [17–22]. Furthermore, it is essential to identify additional markers for improved assessment. Scherpenisse et al. [23] found a potential predictive marker of immunological failure, the cell-associated HIV-1 unspliced-to-multiply-spliced (US/MS) RNA ratio, which was positively correlated with markers of CD4^+^ T cell activation and apoptosis during ART treatment; the higher the US/MS RNA ratio the higher the frequency of HIV-infected cells, leading to sustained immune activation and apoptosis, resulting in decreased immune response to ART.

In clinic, a single index is often inadequate to independently predict disease progression with satisfactory results. However, the combination of several single indexes may greatly improve the predictive effect. Medical nomograms based on various markers have been increasingly used in oncology and other areas of medicine in recent years. In addition, multiple prognostic models for PLWH have been established [24, 25]. However, scoring models for predicting the risk of incomplete immune reconstitution in China have not been reported. Since several risk factors have been identified for INRs, a specific model is needed to predict poor immune reconstitution in advance. Thus, this study aimed to select potential indicators to construct a predictive model based on multivariate logistic regression analysis, providing improved prevention and individualized treatment in PLWH who are at high risk of poor immune reconstitution at the time of primary treatment.

Then, a unique scoring system was created using the primary predictive model's modified nomogram for easy clinical application. Additionally, in a retrospective analysis, we internally verified the diagnostic capabilities of the improved scoring model.

## Methods

### Population and study design

This was a retrospective study of data collected from the China’s for Disease Prevention and Control (CDC)’s Information System. Patients with HIV/AIDS treated at Wuhan Jinyintan Hospital from December 2006 to October 2020 were included for the purpose of model construction and internal validation. Those Participants were splited randomly into the training and internal validation sets in a ratio of 7:3. Besides, the external validation set was obtained from Huangshi and Jingzhou, covering the period from May 2015 to May 2016. Inclusion criteria were: (1) Complete laboratory test confirming HIV infection; (2) Treatment with a combination ART regimen containing at least three drugs; (3) With follow-up results after two years of ART; (4) Age > 15 years. Exclusion criteria were: (1) Previous exposure to ART; (2) VL ≥ 400 copies/mL after 24 months of ART, indicating virologic treatment failure [26]. INR defined as the total CD4^+^ T cell counts < 350 cells/µL at 2 years after cART initiation, with an undetectable plasma VL(< 50 copies/mL) [27].

### Data collection

Demographic characteristics, clinical data and laboratory indexes were collected, including age at the time of diagnosis, sex, body mass index (BMI) calculated as weight/height^2^ (kg/m^2^), infection route, marital status, interval from diagnosis to ART, WHO clinical stage of the HIV disease, opportunistic infection (OI), coinfection with other bacteria or virus, several clinical symptoms, tumors, ART regimens, CD4^+^ T cells, VL, white blood cells (WBC), platelets (PLT), hemoglobin (HB), alanine aminotransferase (ALT), aspartate transaminase (AST), total bilirubin (TBIL), serum creatinine (Scr), triglycerides (TG), serum total cholesterol (TC) and blood glucose (BG). These parameters were obtained by trained professionals every 3 months.

### Data processing

There is no straightforward way to determine the right sample size for a multivariate regression model. A predictive component requires at least 10 effective outcomes, according to previous reports, based on a cautious estimate [28].

**Fig. 1:**
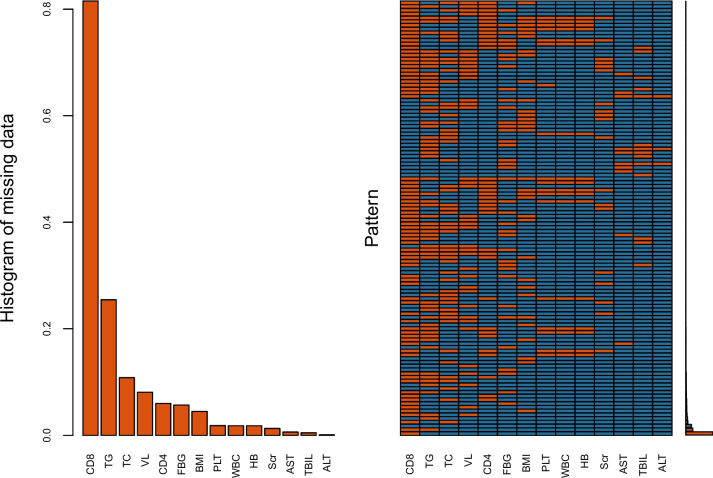
Proportion and distribution pattern of missing values in training set. Abbreviations: BMI, body mass index; CD4: CD4^+^ T lymphocyte; VL: viral load; WBC: white blood cell; PLT: platelet; Hb: hemoglobin; Scr: serum creatinine; TG: triglyceride; TC: total cholesterol; FBG: fasting blood-glucose; ALT: alanine aminotransferase; AST: aspartate aminotransferase; TBIL: total bilirubin

Multiple imputations were used to acquire suitable values for missing data before data analysis since directly discarding data with missing values might cause selection bias or decrease the power of a test. The missing values of the training set were analyzed (Fig. 1). The present study also conducted a sensitivity analysis to assess the impact of imputation of missing values. (sTable 1).

### Statistical analysis

Variables in the training and validation sets were described as number (percentage) or median (interquartile range, IQR), as appropriate. Continuous variables among groups were compared by the Mann–Whitney U test. Meanwhile, categorical variables were compared by the chi-square test, the fisher’s exact test or Wilcoxon rank sum test.

Univariate logistic regression analysis (ULRA) was carried out to select factors in the training set. Then, 34 potential variables with *P* < 0.1 were retained for further analysis. After multivariate logistic regression, 15 candidate predictors were retained. Variables were further selected considering statistically significant parameters and medically important parameters such as availability at first assessment and objectivity of the metric. Finally, five variables, extracted by experienced physicians, were included in the predictive model with the highest predictive performance.

### Presentation of the nomogram

Based on the five most significant variables, a nomogram model with an appropriate predictive ability was developed. The discrimination and calibration of the predictive model was evaluated to test the effectiveness of the model. Among the training set, internal validation, and external validation sets, receiver operating characteristic (ROC) curve analysis was utilized to quantify the discriminative value of the model, and a calibration curve was used to evaluate the calibration. Furthermore, decision curve analysis (DCA) was used to evaluate the clinical utilities of the model.

Data analysis used SPSS version 26.0 (IBM Inc., Chicago, IL, USA) and R-Studio for windows (version 4.2.0) (http://cran.r-project.org). Two-sided *p* < 0.05 was considered statistically significant.

## Results

According to the above inclusion and exclusion criteria, 3783 participants were confirmed and followed up in Wuhan Jinyintan Hospital from 2006 to 2020, and 878 participants in other cities of Hubei from 2015 to 2016. We divided them into three groups, 2678 in the training set, 1105 in the internal validation set, and 878 in the external validation set. A detailed flow diagram of participants selection is presented in sFig. 1. The characteristics of the training and validation sets were presented in Table 1.

**Table 1:** Baseline characteristics of the training set and validation sets

Variables	Derivation set	Internal validation set	External validation set
**Age at ART initiation (years)**	33 (25,49)	32 (25,48)	41 (28,51)
**Sex(%)**			
Male	2429(90.7)	1005(91.0)	716(81.5)
Female	249(9.3)	100(9.0)	162(18.5)
**Marital status(%)**			
Married	786(29.4)	312(28.2)	367(41.8)
Unmarried	1892(70.6)	793(71.8)	511(58.2)
**Route of HIV exposure(%)**			
MSM	1785(66.7)	764(69.1)	522(59.5)
Heterosexual transmission	851(31.8)	334(30.2)	345(39.3)
Injection drug use	18(0.7)	3(0.3)	4(0.5)
Blood transfusion	3(0.1)	0(0)	3(0.3)
Others	21(0.8)	4(0.4)	4(0.5)
**Coinfection(%)**			
HBsAg +	234(8.7)	105(9.5)	61(6.9)
Anti HCV +	54(2.0)	27(2.4)	9(1.0)
Herpes Zoster	108(4.0)	45(4.1)	30(3.4)
PCP	72(2.7)	27(2.4)	17(1.9)
Pulmonary infection	138(5.2)	62(5.6)	10(1.1)
tumor	12(0.4)	3(0.3)	4(0.5)
**WHO stage(%)**			
I	574(21.4)	222(20.1)	504(57.4)
II	1306(48.8)	559(50.6)	151(17.2)
III	616(23.0)	245(22.2)	167(19.0)
IV	182(6.8)	79(7.1)	56(6.4)
**ART initiation regimen(%)**			
AZT + 3TC + NVP/EFV	986(36.8)	375(33.9)	0.093
D4T + 3TC + NVP/EFV	25(0.9)	10(0.9)	0.934
TDF + 3TC + NVP/EFV	1570(58.6)	674(61.0)	0.177
TDF + 3TC + LPV/r	18(0.7)	10(0.9)	0.447
Other	79(2.9)	36(3.3)	0.616
**ART delay**	1.4(0.9,2.6)	1.4(0.9,2.7)	1.4(0.6,6.6)
**BMI, kg/m**	21.5(19.6,23.4)	21.3(19.6,23.4)	21.0(19.1,22.9)

*Abbreviations*: *MSM* men who have sex with men, *PCP* pneumocystis carinii pneumonia, *BMI* body mass index, *AZT* zidovudine, *3TC* lamivudine, *NVP* nevirapine, *EFV* efavirenz, *D4T* stavudine, *TDF* tenofovir disoproxil, *LPV/r* lopinavir/ritonavir

Of all PLWH, 20.5% (955/3782) were INRs, including 21.8% (583/2678) in the training set, 22.0% (243/1105) in the internal validation set, and 14.7% (129/878) in the external validation set.

**Fig. 2:**
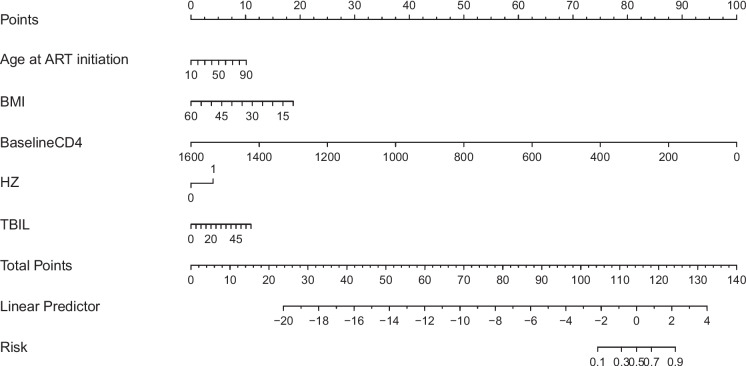
Nomogram of predictors for predicting incomplete immune reconstitution of PLHIV. Abbreviations: BMI, body mass index; CD4: CD4^+^ T lymphocyte; HZ: Herpes zoster; TBIL: total bilirubin

**Fig. 3:**
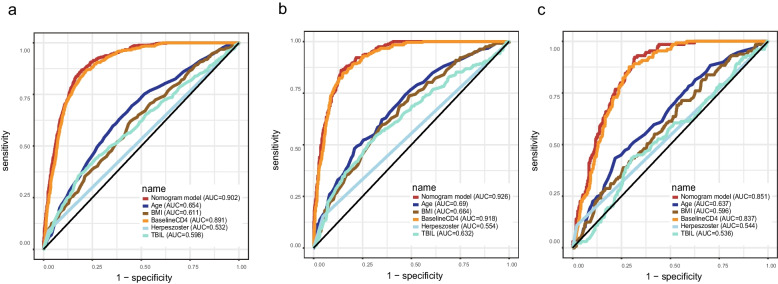
ROC curves of age, baseline CD4, BMI, HZ, TBIL and nomogram in the training set (**a**), the internal validation set (**b**) and the external validation set (**c**). Abbreviations: BMI, body mass index; CD4: CD4^+^ T lymphocyte; HZ: Herpes zoster; TBIL: total bilirubin

**Fig. 4:**
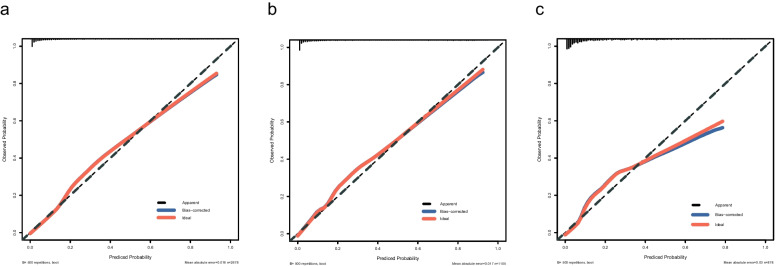
Calibration curves for predicting incomplete immune reconstitution of PLHIV in the training set (**a**), the internal validation set (**b**) and the external validation set (**c**). Abbreviations: BMI, body mass index; CD4: CD4^+^ T lymphocyte; HZ: Herpes zoster; TBIL: total bilirubin

Based on the ULRA of the training set, 34 factors were significantly associated with the INRs (sTable 2). In addition, variables with *p* < 0.1 were selected by experienced physicians for further multivariate logistic regression analysis. Finally, five predictors (baseline CD4, age at the initiation of ART, BMI, Herpes zoster and TBIL) were selected as independent risk factors for the INR status (Table 2). Hence, utilizing these five predictors, we developed a nomogram model (Fig. 2), tested the discriminative power and calibration of the predictive model. The individual and combined performances of these five factors were subsequently assessed comprehensively through ROC analysis. The AUCs for the nomogram model, age, BMI, CD4, HZ and TBIL were 0.902, 0.654, 0.611, 0.891, 0.532, 0.598 in training set (Fig. 3a); 0.926, 0.690, 0.664, 0.918, 0.552, 0.632 in internal validation set (Fig. 3b); and 0.851, 0.637, 0.596, 0.837, 0.544, 0.536 in external validation set (Fig. 3c), respectively. The calibration curves for train and validation sets showed no statistically significant variation from a perfect match between the predicted and actual values (Fig. 4).

**Table 2:** Five significant parameters in the multivariate regression analysis

Variable	β Coefficient	Standard Error	OR (95%CI)	*P*-value
Age at ART initiation	0.028	0.004	1.028(1.020–1.037)	< 0.001
BMI	-0.083	0.022	0.921(0.883–0.960)	< 0.001
Baseline CD4	-0.014	0.001	0.986(0.985–0.988)	< 0.001
HZ	0.894	0.271	2.446(1.437–4.161)	0.001
TBIL	0.040	0.012	1.041(1.017–1.066)	0.001

*Abbreviations*: *BMI* body mass index, *CD4* CD4^+^ T lymphocyte, *HZ* Herpes zoster, *TBIL* total bilirubin

**Fig. 5:**
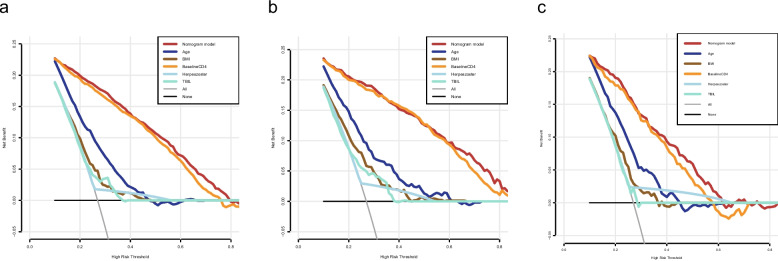
The DCA curves of age, baseline CD4, BMI, HZ, TBIL and nomogram in the training set (**a**), the internal validation set (**b**) and the external validation set (**c**). Abbreviations: BMI, body mass index; CD4: CD4^+^ T lymphocyte; HZ: Herpes zoster; TBIL: total bilirubin

The decision curve analysis also indicated that the nomogram was feasible to make valuable and beneficial judgments in clinical setting. As depicted in Fig. 5a-c, clinical applications using the developed nomograms yielded better clinical benefits within a threshold probability of 0.1 to 0.8, both in the training and validation sets.

Furthermore, to facilitate the application of the predictive model in clinic, dynamic nomograms were constructed as online scoring systems, which are available at https://husteryjs.shinyapps.io/INRs_prediction/.

## Discussion

Despite virological response, INRs have significantly decreased peripheral CD4^+^ T cell count and functionality after at least 1 ~ 2 years of ART [6, 29]. Patients with poor immune status experience chronic immune activation, resulting in higher risks of OIs, malignancies and other nADE [30]. Among all participants, the number of INRs was 826, accounting for 21.8%, including 21.8% and 22.0% in the training and validation sets, respectively. These outcomes corroborated a previous study that found a percentage of INRs in PLWH of 15–30% [31]. For early diagnosis and treatment, in this study, we developed and validated a feasible and simple visual nomogram as a new approach for predicting the development of immune recovery.

The novel approach combines several prominent parameters to create a predictive model for improved diagnosis. This predictive model was constructed based on the derivation and validation cohorts, in which risk factors were selected though logistic regression and their risk scores were evaluated based on the stepwise regression model. A predictive model was developed in the derivation cohort, containing 5 variables: baseline CD4, age at the initiation of ART, BMI, HZ and TBIL. Then, the validation set was applied to assess the efficacy of the predictive model.

In the training and validation sets, the developed nomogram demonstrated good calibration and had high diagnostic performance and clinical utility. We transformed the nomogram into an online calculator to help physicians in clinical practice.

The five parameters utilized in this study were significantly associated with higher risks of failed acquisition of sufficient immune restoration in PLWH, and have important clinical implications.

CD4^+^ T cell count and VL are commonly considered the important markers of treatment outcomes, with associations with long-term prognosis, as well as influencing indicators of immune reconstitution. Indeed, mounting evidence [22, 32–34] shows low baseline CD4^+^ T cell count negatively impacts long-term CD4^+^ T cell recovery in both amount and functionality, affecting the progression of HIV infection. In addition, Jiang et al. [35] found that baseline HIV VL is significantly associated with CD4 + T cell restoration among PLWH. However, because we did not include the baseline VL but choose VL after 2 years treatment, no matched conclusion was obtained from our data.

Previous findings [32, 36, 37] indicate older age may be a risk factor for incomplete CD4^+^ T cell recovery in PLWH, suggesting age may exert a strong effect on long-term recovery of CD4^+^ T cells. This was also found in the newly developed model, showing that median maximal CD4^+^ T cell count is higher in patients aged 16–32.5 years than in patients aged 32.5 years after ART treatment.

Furthermore, this study suggested that male patients have higher risk of poor immune reconstitution and mortality than females. This is common with the finding of other studies, this may be due to the fact that female patients receive HAART at an early stage, reducing the incidence of opportunistic infections as well as achieving rapid CD4 cell recovery. In addition, traced back to differences in life customs, metabolism and adherence between males and females [38, 39]. Kroeze et al. [40] corroborated the above literature data.

In addition, some OIs can also be considered predictors of immune reconstitution. HZ is caused by a variety of diseases that affect immunity, and its incidence increases with decreasing immune levels [41, 42]. Hawkins et al. [43] and Zou et al. [44] found that PLWH with lower CD4 and unsuppressed HIV-1 RNA have the higher risk of HZ. Therefore, we speculate that HZ represents a manifestation of immune suppression, to some extent.

It has been shown that patients with high pre-treatment BMI have a substantial gain in CD4^+^ T lymphocyte recovery independently [45, 46]. This may be because BMI contributes to some extent to drug metabolism, thus affecting the efficacy of cART.

TBIL is mostly produced by destroyed red blood cells, somewhat reflecting the liver function of an individual [47]. In the present study, a negative correlation was found between TBIL and immune recovery in PLWH. Some study [48, 49] found higher HIV RNA and longer duration of HIV viremia were the independent factors to increased risk of HCC, and the presence of liver damage was substantially linked with HIV-1 viral load. While no prior investigation has explicitly illuminated this observation, our supposition is that abnormal liver function could potentially affect the metabolism and absorption of ART drugs, which may decrease treatment efficacy and affect disease progression. However, further investigation is requisite to test the above hypothesis.

Previous findings [50] indicate that the timing of ART initiation also affects long-term immune recovery, regardless of the selected ART regimen. Since 2016, WHO recommends that once diagnosed, all HIV-infected patients should start ART, regardless of CD4 cell count [51]. Engsig et al. suggested that prolonged immunological suppression is a risk factor for incomplete CD4^+^ cell recovery in patients with otherwise successful HAART [32]. However, we did not observe the same outcome in the current cohort, which might be because the participants examined were recently diagnosed cases. Jain et al. proposed that immune restoration may enhance the rate of HBsAg clearance in HIV patients [52]. This means co-infection with other viruses such as Hepatitis B virus (HBV) is another strong risk factor for suboptimal immune recovery, although the underlying mechanism is not fully elucidated, and this notion was not confirmed by our current data.

The present study had several advantages. First, the above model was based on a retrospective cohort with a large sample size, as the first predictive model assessing the risk of becoming INR in an early stage, which showed good performance in an independent validation dataset, and rigorously adhered to known guidelines (TRIPOD) for model construction and validation. Secondly, this model performed well in the validation set, which suggests its potential generalizability. Thirdly, this model can more accurately help clinicians make decisions, with a high AUC. Fourthly, we developed a user-friendly online calculator that only requires the input of a few parameters, and all data conversions and computations are built right into the system, in order to decrease the difficulties imposed by model complexity in clinical application.

We developed and validated a model consisting of 5 clinical and laboratory variables for accurate prediction of the risk of poor immune reconstitution at the time of primary diagnosis. This model can help predict disease progression and regression, providing efficient and precise treatments to improve the life expectancy and quality of life of patients.

This study also had several limitations. First, CD8^+^ T cell count was not included as a candidate predictor in our model due to its high percentage of missing values. This was largely caused by the inherent drawback of retrospective data collection. Next, there was a bias in the predicted accuracy. Even though the majority of indicators in the developed model may be assessed objectively, the route of HIV acquisition is reported by the patients themselves, which could be biased.

In conclusion, A sophisticated nomogram model has been developed and externally validated for the prediction of the risk of poor immune reconstitution at the time of primary diagnosis in this study. we recommend the widespread application of the novel nomogram model to effectively and efficiently identify individuals who are at a heightened risk of INR.

### Supplementary Information

**Additional file 1: Supplementary table 1.** Sensitivity analysis in imputation for missing data. **Supplementary table 2.** Univariable and multivariable logistic regression analysis of the training set. **Supplementary Figure 1.** Flow diagram of participants selection. Abbreviations: INRs, immunologic non responders; VL, viral load ; ART, antiretroviral therapy.

## Data Availability

The datasets used and/or analysed during the current study are available from the corresponding author on reasonable request.
