# Supplementary material for: Incomplete immune reconstitution and its predictors in people living with HIV in Wuhan, China
Source: BMC Public Health. 2023 Sep 16;23:1808. doi: 10.1186/s12889-023-16738-w (PMC10505310; doi:10.1186/s12889-023-16738-w)
Supplement: Supplementary file 1 — Additional file 1: Supplementary table 1. Sensitivity analysis in imputation for missing data. Supplementary table 2. Univariable and multivariable logistic regression analysis of the training set. Supplementary Figure 1. Flow diagram of participants selection. Abbreviations: INRs, immunologic non responders; VL, viral load ; ART, antiretroviral therapy. [file 12889_2023_16738_MOESM1_ESM.docx]

**Supplementary table 1** Sensitivity analysis in imputation for missing data

| **Variable** | **Imputation after** | **Imputation before** | **P-value** |
| --- | --- | --- | --- |
| BMI | 21.40(19.50,23.40) | 21.50(19.60,23.40) | 0.350 |
| CD4 | 267.0(149.0,386.0) | 272.0(158.5,391.0) | 0.177 |
| VL | 38797.0(9616.0,120000.0) | 40312.0(10822.0,120920.0) | 0.268 |
| WBC | 4.88(3.96,5.96) | 4.91(3.99,6.00) | 0.240 |
| PLT | 191.0(155.0,230.0) | 192.0(156.0,230.0) | 0.550 |
| Hb | 143.0(129.0,152.0) | 143.0(130.0,152.0) | 0.247 |
| Scr | 72.6(64.0,82.0) | 72.4(64.0,82.1) | 0.937 |
| TG | 1.29(0.91,1.87) | 1.26(0.90,1.84) | 0.328 |
| TC | 3.86(3.34,4.40) | 3.84(3.33,4.38) | 0.374 |
| FBG | 5.40(4.97,6.00) | 5.40(4.94,6.00) | 0.243 |
| ALT | 22.0(15.0,33.0) | 22.0(15.0,33.0) | 0.461 |
| AST | 24.0(20.0,31.0) | 24.0(20.0,31.0) | 0.369 |
| TBIL | 11.2(8.5,14.7) | 11.2(8.5,14.8) | 0.915 |

**Abbreviations: BMI**, body mass index; **CD4**: CD4^+^ T lymphocyte; **VL**: viral load; **WBC**: white blood cell; **PLT**: platelet; **Hb**: hemoglobin; **Scr**: serum creatinine; **TG**: triglyceride; **TC**: total cholesterol; **FBG**: fasting blood-glucose; **ALT**: alanine aminotransferase; **AST**: aspartate aminotransferase; **TBIL**: total bilirubin.

**Supplementary table 2** Univariable and multivariable logistic regression analysis of the training set.

| Parameter |  | **Univariable** | | **Multivariable** | |
| --- | --- | --- | --- | --- | --- |
|  |  | OR (95%CI) | P value | OR (95%CI) | P value |
| Age at HIV diagnosis |  | 1.035(1.029-1.041) | **<0.001** |  |  |
| **Age at ART initiation** |  | 1.035(1.029-1.042) | **<0.001** | 1.028(1.020-1.037) | <0.001 |
| Gender | male | Ref |  |  |  |
|  | female | 1.273(0.943-1.719) | 0.115 |  |  |
| Married | No | Ref |  |  |  |
|  | Yes | 2.142(1.770-2.594) | **<0.001** |  |  |
| Injection drug use | No | Ref |  |  |  |
|  | Yes | 1.805(0.675-4.830) | 0.240 |  |  |
| MSM | No | Ref |  |  |  |
|  | Yes | 0.439(0.364-0.530) | **<0.001** |  |  |
| Heterosexual | No | Ref |  |  |  |
|  | Yes | 2.207(1.827-2.665) | **<0.001** |  |  |
| Blood transfusion | No | Ref |  |  |  |
|  | Yes | 7.208(0.652-79.634) | 0.107 |  |  |
| Opportunistic infection | None | Ref |  |  |  |
|  | 1 | 2.525（2.041-3.125） | **<0.001** |  |  |
|  | ≥2 | 2.975（2.214-3.998） | **<0.001** |  |  |
| Skin damage | No | Ref |  |  |  |
|  | Yes | 1.778(1.289-2.453) | **<0.001** |  |  |
| Fever for more than 1m | No | Ref |  |  |  |
|  | Yes | 2.279(1.694-3.064) | **<0.001** |  |  |
| Diarrhea for more than 1m | No | Ref |  |  |  |
|  | Yes | 0.865(0.543-1.377) | 0.54 |  |  |
| **Herpes zoster** | No | Ref |  |  |  |
|  | Yes | 3.709(2.513-5.474) | **<0.001** | 2.446(1.418-4.219) | 0.001 |
| PCP | No | Ref |  |  |  |
|  | Yes | 6.406(3.939-10.420) | **<0.001** |  |  |
| Pulmonary infection | No | Ref |  |  |  |
|  | Yes | 4.470(3.156-6.331) | **<0.001** | 0.592(0.378-0.927) | 0.022 |
| Tumor | No | Ref |  |  |  |
|  | Yes | 1.199(0.324-4.443) | 0.786 |  |  |
| Symptoms | None | Ref |  |  |  |
|  | 1 | 1.222(0.969-1.541) | **0.09** |  |  |
|  | ≥2 | 1.668(1.312-2.120) | **<0.001** |  |  |
| Fever | No | Ref |  |  |  |
|  | Yes | 1.580(1.186-2.105) | **0.002** | 0.595(0.401-0.882) | 0.010 |
| Cough | No | Ref |  |  |  |
|  | Yes | 1.894(1.481-2.422) | **<0.001** |  |  |
| Night sweats | No | Ref |  |  |  |
|  | Yes | 1.521(1.115-2.076) | **0.008** |  |  |
| Diarrhea | No | Ref |  |  |  |
|  | Yes | 0.667(0.434-1.025) | **0.065** | 0.497(0.285-0.865) | 0.013 |
| Rash | No | Ref |  |  |  |
|  | Yes | 1.696(1.186-2.426) | **0.004** |  |  |
| Lymph node swelling | No | Ref |  |  |  |
|  | Yes | 0.557(0.374-0.828) | **0.004** | 0.461(0.284-0.746) | 0.002 |
| WHO clinical stage | 1 | Ref |  |  |  |
|  | 2 | 1.607(1.169-2.207) | **0.003** |  |  |
|  | 3 | 5.507(3.988-7.603) | **<0.001** |  |  |
|  | 4 | 14.753(9.816-22.171) | **<0.001** |  |  |
| **BMI** |  | 0.876(0.847-0.906) | **<0.001** |  |  |
| Treatment delay |  | 0.996(0.989-1.004) | 0.337 |  |  |
| AZT+3TC+NVP/EFV | No | Ref |  |  |  |
|  | Yes | 0.948(0.783-1.147) | 0.583 |  |  |
| D4T+3TC+NVP/EFV | No | Ref |  |  |  |
|  | Yes | 6.540(2.875-14.879) | <0.001 |  |  |
| TDF+3TC+NVP/EFV | No | Ref |  |  |  |
|  | Yes | 0.975(0.810-1.175) | 0.791 |  |  |
| TDF+3TC+LPV/r | No | Ref |  |  |  |
|  | Yes | 1.027(0.337-3.132) | 0.963 |  |  |
| Other | No | Ref |  |  |  |
|  | Yes | 0.838(0.474-1.482) | 0.543 |  |  |
| **Baseline CD4** |  | 0.986(0.985-0.987) | **<0.001** | 0.985(0.984-0.987) | <0.001 |
| Baseline VL |  | 1.000(1.000-1.000) | 0.167 |  |  |
| Baseline VL(log) |  | 1.135(1.052-1.225) | **0.001** |  |  |
| HBsAg | No | Ref |  |  |  |
|  | Yes | 1.941(1.452-2.594) | **<0.001** |  |  |
|  | Unknow | 1.153(0.802-1.658) | 0.442 |  |  |
| Anti-HCV | No | Ref |  |  |  |
|  | Yes | 2.002(1.136-3.530) | **0.016** |  |  |
|  | Unknow | 1.143(0.804-1.625) | 0.457 |  |  |
| WBC |  | 0.701(0.655-0.750) | **<0.001** |  |  |
| PLT |  | 0.995(0.993-0.997) | **<0.001** |  |  |
| HB |  | 0.963(0.958-0.968) | **<0.001** |  |  |
| Scr |  | 0.998(0.993-1.004) | 0.497 |  |  |
| TG |  | 1.000(0.967-1.034) | 1.000 |  |  |
| TC |  | 0.851(0.761-0.952) | **0.005** |  |  |
| BG |  | 0.994(0.945-1.047) | 0.828 |  |  |
| ALT |  | 1.001(0.999-1.004) | 0.318 |  |  |
| AST |  | 1.006(1.002-1.010) | **0.002** |  |  |
| **TBIL** |  | 0.946(0.928-0.965) | **<0.001** | 1.037(1.013-1.062) | 0.003 |

**Abbreviations**: **MSM**, men who have sex with men; **PCP**, pneumocystis carinii pneumonia; **AZT**, zidovudine; **3TC**, lamivudine; **NVP**, nevirapine; **EFV**, efavirenz; **D4T**, stavudine; **TDF**, tenofovir disoproxil; **LPV/r**, lopinavir/ritonavir; **WBC**: white blood cell; **PLT**: platelet; **Hb**: hemoglobin; **Scr**: serum creatinine; **TG**: triglyceride; **TC**: total cholesterol; **BG**: blood glucose; **ALT**: alanine aminotransferase; **AST**: aspartate aminotransferase; **TBIL**: total bilirubin.


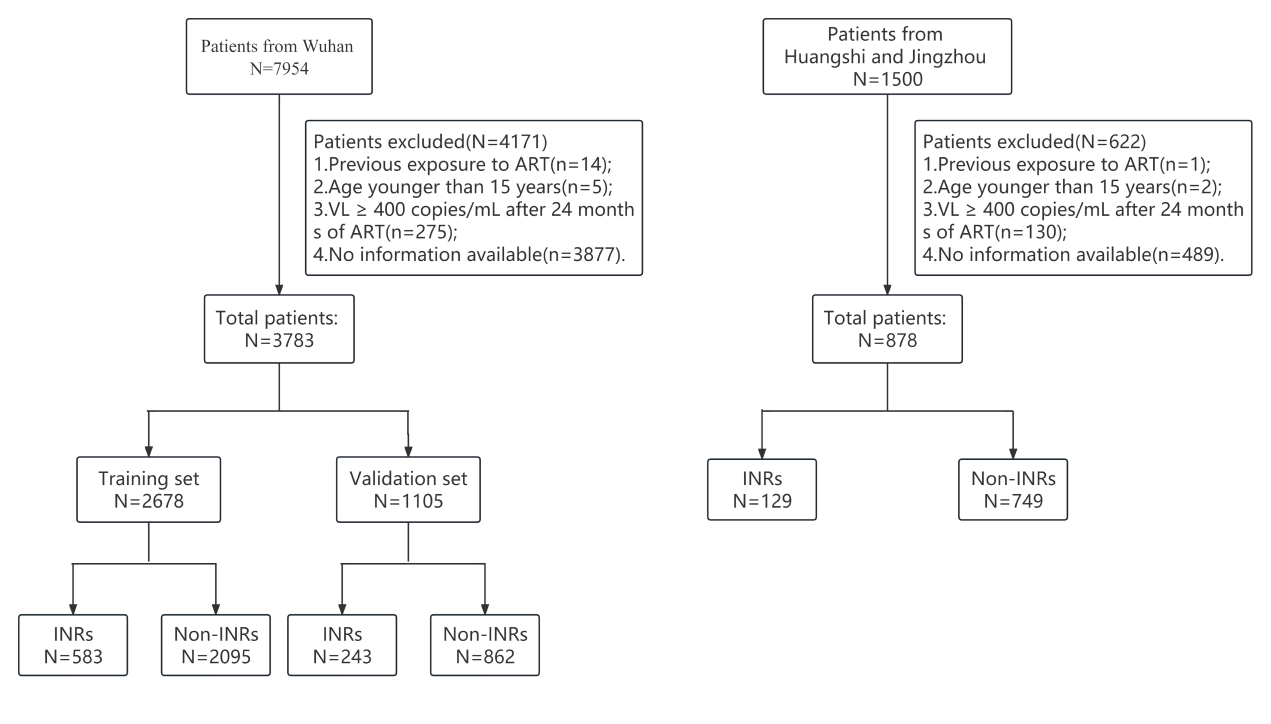


**Supplementary Figure 1**. Flow diagram of participants selection. **Abbreviations**: **INRs**, immunologic non responders; **VL**, viral load ; **ART**, antiretroviral therapy.
